# Pancreatic Duct Obstruction in a Middle-Aged Woman: A Case Report

**DOI:** 10.1089/pancan.2016.0019

**Published:** 2017-02-01

**Authors:** Mustafa Suker, Michael Doukas, Casper van Eijck, Katharina Biermann

**Affiliations:** ^1^ Department of Surgery, Erasmus MC, University Medical Center Rotterdam, Rotterdam, The Netherlands.; ^2^ Department of Pathology, Erasmus MC, University Medical Center Rotterdam, Rotterdam, The Netherlands.

**Keywords:** granular cell tumor, pancreatic neoplasms, pancreatic duct dilatation

## Abstract

**Background:** Granular cell tumors (GCTs) are rare benign neoplasms of Schwann cells. These tumors have been described in almost every human organ. Although GCT has been described in the pancreas previously, we present a case report about GCTs in multiple organs at a simultaneous time.

**Case Presentation:** A 51-year-old Caucasian female known with epilepsy and COPD presented with recurrent abdominal pain. Previously, endoscopic mucosal resection in the esophagus and lumpectomy of the right breast were performed for what proved to be GCTs. Computed tomography showed a hypodense unclearly demarcated tumor of the pancreas tail–body with the impression of infiltrative growth and pancreatic duct dilation. The patient underwent an uncomplicated distal pancreatectomy with the pathological examination showing a fibrotic area of 6 mm consisting of diffusely spread nests of large cells embedded in a collagenous stroma of the pancreatic tail. The tumor nuclei were not atypical and the cytoplasm was granular and eosinophilic. The cell clusters stained positive for S-100 and CD68 in the cell cytoplasm. The diagnosis, GCT of the pancreas, was made and the postoperative course was uneventful for our patient, and a year after surgery, there have been no new tumorous lesions detected.

**Conclusion:** We present a rare case of multiple GCTs affecting the breast, esophagus, and pancreas. Although GCT of the pancreas is a rare disease, the diagnosis should be considered if there is GCT in the medical history of the patient.

## Introduction and Background

Granular cell tumor (GCT) was first described by Abrikossoff et al. as a tumor of the tongue, but was later also detected in many other organs.^1^ GCT can occur at any age, and as it varies in location, the presentation is often atypical. Originally, GCT was considered as skeletal muscle origin due to the histologic similarity to myogenic cells. However, the current concept is that GCT is a mesenchymal tumor with a neurogenic origin due to typical positivity for CD68 and S-100.^2^ Although GCT is predicted to exhibit a benign biological behavior, it can infiltrate into the surrounding tissue. It is the infiltrative feature that can give the impression of malignancy on imaging. We present a rare case of GCT in the pancreas in a patient known with GCT in the breast and esophagus.

## Presentation of Case

**FIG. 1.**
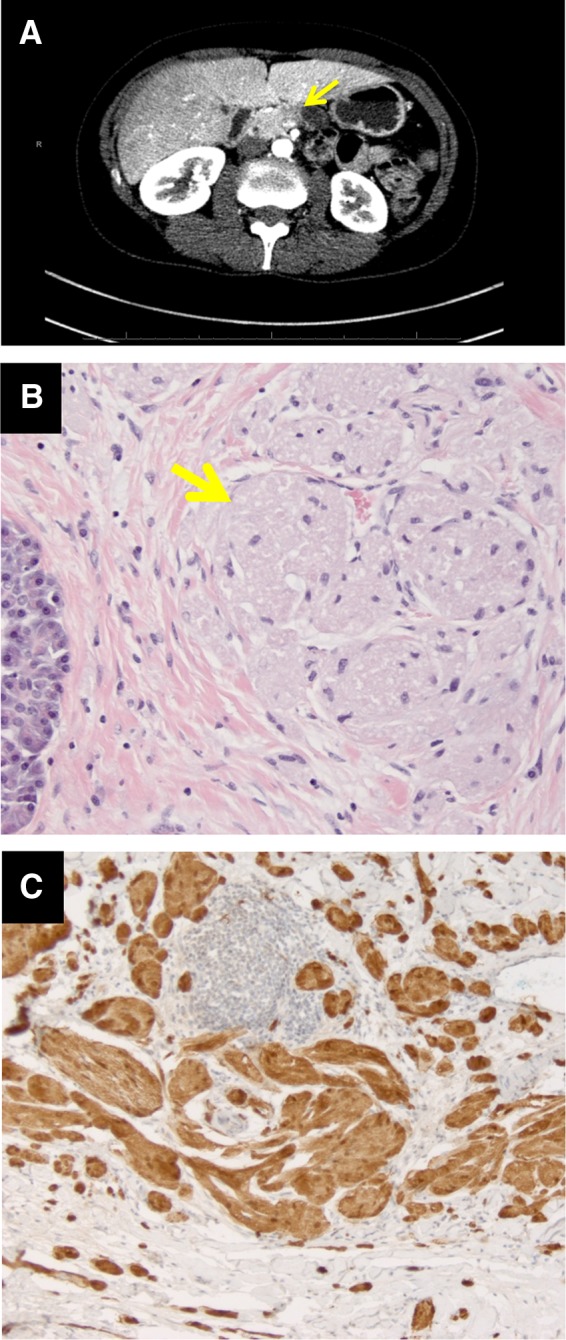
**(A)** Computed tomography scan with the yellow arrow on the pancreatic lesion. **(B)** Hematoxylin and eosin stain × 400 with the yellow arrow on the tumor nuclei. **(C)** Nuclear and cytoplasmic staining with antibodies positive to S-100 protein and cytoplasmic staining positive for CD68 staining × 400.

A 51-year-old Caucasian female known with epilepsy and COPD presented with recurrent abdominal pain. Previously, endoscopic mucosal resection in the esophagus and lumpectomy of the right breast were performed revealing GCTs. Endoscopic ultrasound revealed a marked dilation of the pancreatic duct and next to it a hypoechoic lesion in the pancreas body. Subsequent computed tomography showed a hypodense poorly demarcated tumor of the pancreas in the tail–body with impression of infiltrative growth (Fig. 1A). Common bile duct brush and EUS-guided fine-needle biopsy of the pancreatic tumor were negative for malignancy. The serum tumor marker results were all normal and as follows: Chromogranin A of 67 μg/L (elevated), NSE of 12.4 μg/L, and CA 19.9 of 9 kU/L.

As our clinical working diagnosis was a neuroendocrine tumor, a distal pancreatectomy was performed. Pathological examination of the resection specimen showed a fibrotic area of 6 mm in size consisting of diffusely spread nests of large cells embedded in a collagenous stroma. The tumor nuclei were not atypical and the cytoplasm was granular and eosinophilic (Fig. 1B). These cell clusters stained positive for S-100 and CD68 in the cell cytoplasm (Fig. 1C). All other immunohistochemical markers were negative, including pankeratin, synaptophysin, chromogranin A, CD34, SMA, and melan-A. The entire resection specimen was examined and showed normal pancreatic tissue and three normal lymph nodes. GCT of the pancreas was diagnosed.

After the distal pancreatectomy, the postoperative course was uneventful for our patient, and a year after surgery, there are no new lesions detected.

## Conclusion

To our of knowledge, there are only seven published cases about GCT in the pancreas,^2^ and pancreatic duct obstruction has been described in association with GCT.^3^ In our case, it seems that the patient developed multiple GCTs, which affected the esophagus, mamma, and pancreas.

## Abbreviation Used

GCTs: granular cell tumors
